# Erratum

**DOI:** 10.1590/1518-8345.0000.2792

**Published:** 2016-07-11

Regarding the article "Right to health: (in) congruence between the legal framework and the health system", with DOI number: 10.1590/1518-8345.0995.2679, published in the Rev. Latino-Am. Enfermagem. 2016;24:e2679, page 7:

Where was written:

"Filipa Alexandra Veludo Fernandes

Universidade Católica Portuguesa. Instituto de Ciências da Saúde

Escola Superior Politécnica de Saúde

Caminho da Palma de Cima

1649-023, Lisboa, Portugal

E-mail: fveludo@ics.lisboa.ucp.pt"

Now Read:

"Fernando Mitano

Universidade Lúrio. Campus de Marrere

R. nr. 4250, Km 2,3

Bairro de Marrere

Nampula, Moçambique

E-mail: piqinamita@gmail.com"

Regarding the article "Analgesic efficacy of lidocaine and multimodal analgesia for chest tube removal: A randomized trial study", with DOI number: 10.1590/0104-1169.0498.2642, published in the Rev. Latino-Am. Enfermagem. 2015 Nov.-Dec.;23(6), page 1000:

Where was written:

"Conclusion: the present study suggests that the analgesic effect of the subcutaneous administration of 1% lidocaine combined with multimodal analgesia is most efficacious."

Now Read:

"Conclusion: the present study suggests that the analgesic effect of the subcutaneous administration of 1% lidocaine combined with multimodal analgesia is less effective."

Regarding the article "Drug use, mental health and problems related to crime and violence: cross-sectional study", with DOI number: 10.1590/0104-1169.0478.2663, published in the Rev. Latino-Am. Enfermagem. 2015;23(6):1173-80, page 1173:

Where was written:

"Janet Titus Bourdreaux"

Now Read:

"Janet C. Titus"

Regarding the article "Potential access to primary health care: what do the data from the National Program for Access and Quality Improvement show?", with DOI number: 10.1590/0104-1169.1069.2672, published in the Rev. Latino-Am. Enfermagem. 2016;24:e2672, page 1:

Where was written:

"Severina Alice da Costa Uchôa^1^

Ricardo Alexandre Arcêncio^2^

Inês Santos Estevinho Fronteira^3^

Ardigleusa Alves Coêlho^4^

Claudia Santos Martiniano^4^

Isabel Cristina Araújo Brandão^5^

Mellina Yamamura^6^

Renata Melo Maroto^7^"

Now Read:

"Severina Alice da Costa Uchôa^1^

Ricardo Alexandre Arcêncio^2^

Inês Fronteira^3^

Ardigleusa Alves Coêlho^4^

Claudia Santos Martiniano^4^

Isabel Cristina Araújo Brandão^5^

Mellina Yamamura^6^

Renata Melo Maroto^7^

Anny Karine Freire da Silva^8^"

Where was written:

"Objective: to analyze the influence of contextual indicators on the performance of municipalities regarding potential access to primary health care in Brazil and to discuss the contribution from nurses working on this access. Method: a multicenter descriptive study based on secondary data from External Evaluation of the National Program for Access and Quality Improvement in Primary Care, with the participation of 17,202 primary care teams. The chi-square test of proportions was used to verify differences between the municipalities stratified based on size of the coverage area, supply, coordination, and integration; when necessary, the chi-square test with Yates correction or Fisher's exact test were employed. For the population variable, the Kruskal-Wallis test was used. Results: the majority of participants were nurses (n=15.876; 92,3%). Statistically significant differences were observed between the municipalities in terms of territory (p=0.0000), availability (p=0.0000), coordination of care (p=0.0000), integration (p=0.0000) and supply (p=0.0000), verifying that the municipalities that make up area 6 tend to have better performance in these dimensions. Conclusion: areas 4,5 and 6 performed better in every analyzed dimension, and the nurse had a leading role in the potential to access primary health care in Brazil."

Now Read:

"Objective: to analyze the influence of contextual indicators on the performance of cities regarding potential access to primary health care in Brazil and to discuss the contribution from nurses working on this access. Method: a multicenter descriptive study using secondary data from External Evaluation of the National Program for Access and Quality Improvement in Primary Care, with the participation of 17,202 primary care teams. The chi-square test of proportions was used to verify differences between the cities stratified in the dimensions on size of the coverage group, supply, coordination and integration. When necessary, the chi-square test with Yates correction or Fisher’s exact test were employed. For the population variable, the Kruskal-Wallis test was used. Results: the majority of participants were nurses (n = 15,876; 92.3%). Statistically significant differences were observed between the cities in terms of territory (p=0.0000), availability (p=0.0000), coordination of care (p=0.0000), integration (p=0.0000) and supply (p=0.0000), verifying that the cities that make up group 6 tend to perform better in these dimensions, with a better performance in all dimensions analyzed in groups 4, 5 and 6. Conclusion: weakness in smaller cities, confirming inequities in the potential access to Primary Health Care in Brazil as challenges for universal coverage. The preponderant role of nurses for its achievement is highlighted.

Where was written:

"^1^ Post-doctoral fellow, Instituto de Higiene e Medicina Tropical, Universidade Nova de Lisboa, Lisboa, Portugal. Associate Professor, Departamento de Saúde Coletiva, Universidade Federal do Rio Grande do Norte, Natal, RN, Brazil. Scholarship holder from Conselho Nacional de Desenvolvimento Científico e Tecnológico (CNPq), Brazil.

^2^ PhD, Professor, Escola de Enfermagem de Ribeirão Preto, Universidade de São Paulo, PAHO/WHO Collaborating Centre for Nursing Research Development, Ribeirão Preto, SP, Brazil.

^3^ PhD, Assistant Professor, Instituto de Higiene e Medicina Tropical, Universidade Nova de Lisboa, Lisboa, Portugal.

^4^ PhD, Professor, Departamento de Enfermagem, Universidade Estadual da Paraíba, Campina Grande, PB, Brazil.

^5^ MSc, Professor, Departamento de Enfermagem, Centro Universitário FACEX, Natal, RN, Brazil.

^6^ Doctoral student, Escola de Enfermagem de Ribeirão Preto, Universidade de São Paulo, PAHO/WHO Collaborating Centre for Nursing Research Development, Ribeirão Preto, SP, Brazil. Assistant Professor, Escola de Enfermagem, Universidade Federal do Rio Grande do Norte, Natal, RN, Brazil. Scholarship holder from Fundação de Amparo à Pesquisa do Estado de São Paulo (FAPESP), Brazil.

^7^ Doctoral student, Departamento de Odontologia, Universidade Federal do Rio Grande do Norte, Natal, RN, Brazil."

Now Read:

"^1^ Post-doctoral fellow, Instituto de Higiene e Medicina Tropical, Universidade Nova de Lisboa, Lisboa, Portugal. Associate Professor, Departamento de Saúde Coletiva, Universidade Federal do Rio Grande do Norte, Natal, RN, Brazil. Scholarship holder from Conselho Nacional de Desenvolvimento Científico e Tecnológico (CNPq), Brazil.

^2^ PhD, Professor, Escola de Enfermagem de Ribeirão Preto, Universidade de São Paulo, PAHO/WHO Collaborating Centre for Nursing Research Development, Ribeirão Preto, SP, Brazil.

^3^ PhD, Assistant Professor, Instituto de Higiene e Medicina Tropical, Universidade Nova de Lisboa, Lisboa, Portugal.

^4^ PhD, Professor, Departamento de Enfermagem, Universidade Estadual da Paraíba, Campina Grande, PB, Brazil.

^5^ MSc, Professor, Departamento de Enfermagem, Centro Universitário FACEX, Natal, RN, Brazil.

^6^ Doctoral student, Escola de Enfermagem de Ribeirão Preto, Universidade de São Paulo, PAHO/WHO Collaborating Centre for Nursing Research Development, Ribeirão Preto, SP, Brazil. Assistant Professor, Escola de Enfermagem, Universidade Federal do Rio Grande do Norte, Natal, RN, Brazil. Scholarship holder from Fundação de Amparo à Pesquisa do Estado de São Paulo (FAPESP), Brazil.

^7^ Doctoral student, Departamento de Odontologia, Universidade Federal do Rio Grande do Norte, Natal, RN, Brazil.

^8^ Specialist in Reading and Text Production."

Page 2

Where was written:

"In Brazil, the issue of universal and equitable access has been a concern since the creation of the Unified Health System UHS (SUS) in 1988. This idea is reinforced by the National Policy of Primary Care - BANP (PNAB), in which the potential for access to comprehensive care management through multidisciplinary, interdisciplinary team work is emphasized^(3)^."

Now Read:

"Since the 1988 constitution, Brazil has made efforts towards universal coverage as a right through the Unified Health System (SUS). In 1994, the Family Health Strategy was implemented, based on comprehensive care and multidisciplinary teamwork. Through this strategy, the coverage rate was expanded, reaching 57% of the population (108 million people) in 2013^(3)^."

Where was written:

"Research scenario

In 2012, SUS had 36,361 Basic Health Units (BHU) and 33,404 Family Health Teams (FHT) with coverage in 5,297 municipalities. The adherence to PMAQ occurred with 17,202 Primary Care Teams (PCT). Among these, 16,566 FHT and 636 non- FHT were distributed in 3,944 (70.8%) of the total municipalities, in 14,111 Basic Health Units (BHUs)^(7)^. "

Now Read:

"Research scenario

Adherence to cycle I of the PMAQ amounted to 17,482 Primary Care Teams (PCT), distributed across 3,944 (70.8%) of all cities and 14,111 Basic Health Units (BHUs)^(7)^. In this group, 17,202 were recruited for the study, as their questionnaires were validated in the database of the Ministry of Health."

Pages 3-8:

Classification of municipalities according to the context variables

The municipalities listed in the study are classified into six strata, considering the *per capita* Gross Domestic Product (GDP), the percentage of the population with health insurance, the percentage of the population on the *Bolsa Família* (Family Grant) program, the percentage of the population in extreme poverty, and the population density.

The composition of the extracts considered for each municipality were: the lowest score among the percentage of the population with *Bolsa Família* program, and the percentage of the population in extreme poverty: area 1 - Municipalities with scores lower than 4.82 and a population of up to 10,000 inhabitants; area 2 - Municipalities with scores lower than 4.82 and a population of up to 20 thousand inhabitants; area 3 - municipalities with scores lower than 4.82 and a population of up to 50 thousand inhabitants; area 4 - Municipalities with scores between 4.82 and 5.4, and population of up to 100 thousand inhabitants; area 5 - Municipalities with scores between 5.4 and 5.85, and population of up to 500 thousand inhabitants; and municipalities with a score lower than 5.4, and population between 100 and 500 thousand inhabitants; and area 6 - Municipalities with population over 500,000 inhabitants, or a score less than 5.85^(7)^.

Variables under consideration to evaluate potential access:

The variables considered for evaluating potential access are described in Table 2. The table shows the dimension, characteristic and nature of the variables that are included.

Plan of analysis

Initially, the descriptive analysis of the characteristics area of the municipalities', professional category, and median number of professionals per team was calculated. 

Regarding the performance of municipalities in terms of access, four dimensions of the PMAQ instrument were measured: coverage area, supplies, customer coordination, and integration.

The variables were dichotomized into yes and no. Thereafter, the sum of the responses for each item was calculated, dividing this number by the total sample. To verify differences between the municipalities in relation to the size of potential access, the chi-square test of proportions was used. The chi-square test with Yates or Fisher's exact test correction was applied when necessary. For the population variable, the Kruskal-Wallis test was used to verify differences in relation to the median inhabitants monitored by areas.

After the analysis of the performance of the municipalities within the areas, in relation to access, multivariate statistics by multiple correspondence analyses (MCA) was used, given that the instrument variables were categorical.

 The MCA implementation was based on the steps of Spencer^(13)^ and Mingoti^(14)^, in which the tabulation of responses generated a matrix, with rows corresponding to the participating health professionals, and the columns corresponding to the variables. Subsequently, the matrix turned into a complete disjunctive table (CDT). In the table, the columns represent characteristics of the variables, in which the intersection of Row I with Column J is the *xij*, which is 0 or 1, indicating that the area either has or does not have the characteristic.

The perceptual map was formed by this technique, which is a visual representation of the variables in two or more dimensions. Each variable has a spatial position in the perceptual map, variables perceived as similar or associated are allocated to proximal points on the map, while those not perceived as similar are represented as distal points. The proximity indicates the correspondence between the categories represented in rows and columns of the table.

 The component row or column influences the construction of the axes through its inertia, in relation to the center of gravity. The inertia means the variance of the data set ^(13)^. From the MCA it was possible to extract the most representative dimensions in terms of inertia, which in the study corresponded to the first two. Its contribution to inertia was considered a criterion for selection of the variables.

Results

Table 1 shows characteristics of the sample of 17,202 teams recruited for the study, according to the PMAQ area. The majority of participants were nurses (n =;%), and many of them had less than three years of experience after completing their education.

Among the models of care, in all areas, there was a predominance of the Family Health Strategy (FHS) without oral health. In general, there is a median of one (1) physician, nurse, nursing technicians, and dentist per team. All modalities of care investigated showed that most of the teams did not provide the patient with the opportunity to choose a desired unit for treatment and follow up.

In Table 2, the performance of municipalities in terms of patient access is verified, considering the area established in PMAQ.

Statistically significant differences were identified between the municipalities of area 1, 2 and 3 with area 4, 5 and 6, and the professionals of the last areas had more qualifications (p=0.0000).

Regarding the career plan, no statistically significant difference (p = 0.0000) was observed, and the municipalities of area 4, 5 and 6 had better indicators; lowest values were found in areas 1, 2 and 3. Also, these areas showed statistically significant differences associated with their training policy and continuing education (p=0.0000).

According to Table 2, statistically significant differences in t erms of population coverage were observed, in which area 5 and 6 monitored a median number of people with access well above that of areas 1, 2 and 3. Also, statistically significant differences were present between the municipalities in terms of coverage area (p=0.0000), availability (p=0.0000), coordination of care (p=0.0000), integration (p=0.0000) and supply (p=0.0000), verifying that the municipalities that form area 6 tend to have better performance in these dimensions.

**Table1 -:** Characteristics of study sample, PMAQ Project, Brazil (2012)

**Variables**		**PMAQ Areas**					
		**1**	**2**	**3**	**4**	**5**	**6**
Professional category *n ( %)*							
	Physician	72 (0.42)	59 (0.34)	52 (0.30)	91 (0.53)	143 (0.83)	576 (3.35)
	Nurse	2.058 (11.96)	2.179 (12.67)	2.425 (14.10)	3.119 (18. 13)	2.615 (15.20)	3.480 (20.23)
	Dentist	35 (0.20)	35 (0.20)	50 (0.29)	56 (0.33)	56 (0.33)	101 (0.59)
Years of work/experience *n (%)*							
	Less than 1 year	546 (3.17)	693 (4.03)	801 (4.66)	995 (5.78)	830 (4.83)	875 (5.09)
	Between 1-3 years	867 (5.04)	966 (5.62)	1.068 (6.21)	1.384 (8.05)	1.133 (6.59)	1.598 (9.29)
	Greater than three years	743 (4.32)	608 (3.53)	652 (3.79)	881 (5.12)	843 (4.90)	1.673 (9.73)
	Don´t know/ no response	9 (0.05)	6 (0.03)	6 (0.03)	6 (0.03)	8 (0.05)	11 (0.06)
Type of team *n (%)*							
	Family Health Teams with oral health	1.832 (10.66)	1.798 (10.45)	2.041 (11.86)	2.464 (14.32)	1.767 (10.27)	2.173 (12.63)
	Family Health Teams without oral health	261 (1.52)	398 (2.31)	423 (2.46)	720 (4.19)	942 (5.48)	1.824 (10.60)
	Primary care team with oral health	59 (0.34)	57 (0.33)	45 (0.26)	59 (0.34)	57 (0.33)	51 (0.30
	Primary care teams without oral health	7 (0.04)	9 (0.05)	11 (0.06)	15 (0.09)	43 (0.25)	39 (0.23)
	Others	4 (0.02)	6 (0.03)	4 (0.02)	7 (0.04)	3 (0.02)	66 (0.38)
	Do not Know/No response	2 (0.01)	5 (0.03)	3 (0.02)	1 (0.01)	2 (0.01)	4 (0.02)
Minimum number of physicians in the primary care staff of BHU (n= 16643)							
	Median	1	1	1	1	1	1
	Minimum and Maximum value	0.00 - 4.00	0.00 - 4.00	0.00 - 4.00	0.00 - 11.00	0.00 - 11.00	0.00 - 6.00
Minimum number of nurses in the primary care staff (n=16643)							
	Median	1	1	1	1	1	1
	Minimum and maximum value	0.00 - 4.00	0.00 - 4.00	0.00 - 4.00	0.00 - 4.00	0.00 - 4.00	0.00 - 4.00
Minimum number of dentists in the primary care staff (n=16643)							
	Median	1	1	1	1	1	1
	Minimum and maximum value	0.00 - 6.00	0.00 - 4.00	0.00 - 3.00	0.00 - 6.00	0.00 - 6.00	0.00 - 4.00
Minimum number of nursing technicians in the primary care staff (n=16643)							
	Median	1	1	1	1	1	1
	Minimum and maximum value	0.00 - 13.00	0.00 - 10.00	0.00 - 10.00	0.00 - 8.00	0.00 - 20.00	0.00 - 11.00
Minimum number of nursing assistants in the primary care staff (n=16643)							
	Median	0	0	0	0	0	1
	Minimum and maximum value	0.00 - 9.00	0.00 - 8.00	0.00 - 8.00	0.00 - 8.00	0.00 - 6.00	0.00 - 20.00
Minimum number of dental technicians in the primary care staff (n=16643)							
	Median	0	0	0	0	0	0
	Minimum and maximum value	0.00 - 8.00	0.00 - 8.00	0.00 - 8.00	0.00 - 2.00	0.00 - 3.00	0.00 - 8.00
Minimum number of dental assistants in the primary care staff (n=16643)							
	Median	1	1	1	1	1	0
	Minimum and maximum value	0.00 - 6.00	0.00 - 7.00	0.00 - 8.00	0.00 - 9.00	0.00 - 8.00	0.00 - 10.00
Minimum number of community health workers in the primary care staff (n=16643)							
	Median	6	6	7	6	6	5
	Minimum and maximum value	0.00 - 19.00	0.00 - 50.00	0.00 - 42.00	0.00 - 50.00	0.00 - 56.00	0.00 - 32.00
Allowing the patient to choose team by which he wants to be treated *n (%)*							
	Yes	219 (1.27)	191 (1.11)	180 (1.05)	161 (0.94)	127 (0.74)	303 (1.76)
	No	286 (1.66)	309 (1.80)	303 (1.76)	411 (2.39)	442 (2.57)	1.059 (6.16)
	Not applicable	454 (2.64)	539 (3.13)	516 (3.00)	671 (3.90)	355 (2.06)	196 (1.14)
	Don´t know/No response	1.206 (7.01)	1.234 (7.17)	1.528 (8.88)	2.023(11.76)	1.890 (10.99)	2.599 (15.11)

**Table 2:** - Performance of municipalities on patient access according to the areas, Brazil, 2012

**Dimension**	**Variables**	**PMAQ areas**						
		**1**	**2**	**3**	**4**	**5**	**6**	**p value**
		**n (%)**	**n (%)**	**n (%)**	**n (%)**	**n (%)**	**n (%)**	
Personal qualification	Complementary education (n=17.202)							
	Yes	1.708 (9.93)	1.795 (10.43)	2.050 (11.92)	2.694 (15.66)	2.460 (14.30)	3.642 (21.17)	0.000*
	No	457 (2.66)	478 (2.78)	477 (2.77)	572 (3.33)	354 (2.06)	515 (2.99)	
	Career development programs (n=16.936)							
	Yes	253 (1.49)	159 (0.94)	246 (1.46)	574 (3.39)	581 (3.43)	1.810 (10.69)	0.000*
	No	1.877 (11.08)	2.069 (12.22)	2.245 (13.26)	2.647 (15.63)	2.194 (12.95)	2.279 (13.46)	
	There are continuing education activities involving primary care professionals (n=17.113)							
	Yes	1.432 (8.37)	1.596 (9.33)	1.878 (10.97)	2.601 (15.20)	2.481 (14.50)	3.969 (23.19)	0.000*
	No	720 (4.21)	658 (3.85)	630 (3.68)	650 (3.80)	325 (1.90)	173 (1.01)	
Coverage area	How many people for whom the team is responsible							
	Mean	2165	2273	2527	3266	2814	4157	0.0001^?^
	Risk and vulnerability criteria were considered for defining people for whom the team is responsible (n=15.691)							
	Yes	1.024 (6.53)	1.141 (7.27)	1.323 (8.43)	1.705 (10.87)	1.423 (9.07)	2.648 (16.88)	0.000*
	No	951 (6.06)	877 (5.59)	937 (5.97)	1.265 (8.06)	1.115 (7.11)	1.282 (8.17)	
	There is definition of team coverage area (n=17.150)							
	Yes	2.086 (12.16)	2.197 (12.81)	2.456 (14.32)	3.190 (18.60)	2.763 (16.11)	4.113 (23.98)	0.000*
	No	68 (0.40)	60 (0.35)	63 (0.37)	71 (0.41)	43 (0.25)	40 (0.23)	
	There is a population uncovered by primary care surrounding the team's coverage area (n=17.092)							
	Yes	369 (2.16)	534 (3.12)	888 (5.20)	1.083 (6.34)	1.391 (8.14)	1.513 (8.85)	0.000*
	No	1.783 (10.43)	1.724 (10.09)	1.618 (9.47)	2.170 (12.70)	1.406 (8.23)	2.613 (15.29)	
	How often people from outside the team's coverage area are served by this team (n=16.855)							
	Every day of the week	900 (5.34)	828 (4.91)	1.001 (5.94)	1.247 (7.40)	1.255 (7.45)	2.152(12.77)	0.000*
	Some days of the week	966 (5.73)	1.135 (6.73)	1.201 (7.13)	1.502 (8.91)	1.222 (7.25)	1.673 (9.93)	
	Any day of the week	248 (1.47)	243 (1.44)	266 (1.58)	451 (2.68)	287 (1.70)	178 (1.65)	
Availability	Patients who spontaneously arrive and have their needs heard and assessed (n=17.140)							
	Yes	2.121 (12.37)	2.202 (12.85)	2.442 (14.25)	3.180 (18.55)	2.689 (15.69)	4.078 (23.79)	0.000*
	No	38 (0.22)	59 (0.34)	80 (0.47)	83 (0.48)	108 (0.63)	60 (0.35)	
	The team performs risk and vulnerability assessment in the intake of patients (n=13.739)							
	Yes	1.265 (9.21)	1.385 (10.08)	1.645 (11.97)	2.286 (16.64)	2.050 (14.92)	3.442 (25.05)	0.0066*
	No	192 (1.40)	221 (1.61)	248 (1.81)	324 (2.36)	236 (1.72)	445 (3.24)	
	The schedule is organized to conduct home visitation (n=13.951)							
	Yes	1.418 (10.16)	1.628 (11.67)	1.865 (13.37)	2.391 (17.14)	2.253 (16.15)	3.697 (26.50)	0.000*
	No	134 (0.96)	115 (0.82)	114 (0.82)	149 (1.07)	104 (0.75)	83 (0.590)	
Coordination of care	Keep a record of high risk patients referred to other points of care (n=17.104)							
	Yes	826 b(4.83)	818 (4.78)	1.104 (6.45)	1.474 (8.62)	1.353 (7.91)	2.385 (13.94)	0.000*
	No	1.310 (7.66)	1.439 (8.41)	1.405 (8.21)	1.785 (10.44)	1.449 (8.47)	1.756 (10.27)	
	There is a document proving (n=							
	Yes	605 (7.60)	638 (8.02)	913 (11.47)	1.206 (15.15)	1.132 (14.22)	1.978 (24.85)	0.000*
	No	221 (2.78)	180 (2.26)	191 (2.40)	268 (3.37)	221 (2.78)	407 (5.11)	
	There are protocols that guide the prioritization of cases needing referral (n=17.037)							
	Yes	581 (3.41)	613 (3.60)	807 (4.74)	1.213 (7.12)	1.228 (7.21)	2.907 (17.06)	0.000†
	No	1.558 (9.14)	1.636 (9.60)	1.685 (9.89)	2.036 (11.95)	1.567 (9.20)	1.206 (7.08)	
Integration	There is a regulation center (n=17.201)							
	Yes	1.880 (10.93)	2.006 (11.66)	2.239 (13.02)	2.907 (16.90)	2.540 (14.77)	4.027 (23.41)	0.000*
	No	284 (1.65)	267 (1.55)	288 (1.67)	359 (2.09)	274 (1.59)	130 (0.76)	
	There is a referral form for patients moving to other points of care (n=17.201)							
	Yes	1.752 (10.19)	1.828 (10.63)	2.138 (12.43)	2.970 (17.27)	2.615 (15.20)	4.055 (23.57)	0.0000*
	No	412 (2.40)	445 (2.59)	389 (2.26)	296 (1.72)	199 (1.16)	102 (0.59)	
Supply	Receive enough basic medicines from pharmacy to serve its population (n=17.161)							
	Yes	1.459 (8.50)	1.490 (8.68)	1.722 (10.03)	2.210 (12.88)	1.830 (10.66)	2.898 (16.89)	0.0000*
	No	378 (2.20)	457 (2.66)	614 (3.58)	644 (3.75)	718 (4.18)	2.077 (6.28)	
	Do not receive	316 (1.84)	320 (1.86)	187 (1.09)	406 (2.37)	263 (1.53)	172 (1.00)	
	Offers service of complementary and integrative practices for patients of the area (n=17.199)							
	Yes	235 (1.37)	230 (1.34)	305 (1.77)	381 (2.22)	512 (2.98)	1.546 (8.99)	0.0000*
	No	1.929 (11.22)	2.042 (11.87)	2.222 (12.92)	2.885 (16.77)	2.301 (13.38)	2.611 (15.18)	
	Conducts home visits (n=17.199)							
	Yes	2.146 (12.48)	2.262 (13.15)	2.521 (14.66)	3.253 (18.91)	2.802 (16.29)	4.148 (24.12)	0.0075*
	No	18 (0.10)	10 (0.06)	6 (0.03)	13 (0.08)	11 (0.06)	9 (0.05)	
	The families in the coverage area are visited at intervals according to risk and vulnerability assessment? (n=17.132)							
	Yes	1.963 (11.46)	2.069 (12.08)	2.345 (13.69)	2.997 (15.30)	2.621 (15.30)	3.986 (23.27)	0.0000*
	No	183 (1,07)	193 (1,13)	176 (1,03)	256 (1,49)	181 (1,06)	162 (0,95)	

* p value statistically significant (p<0.05) † Kruskal-Wallis test

When compared by professional category (Table 3), a statistically significant difference is again identified, in which a higher proportion of both physicians as well as dentists tend to refer to more positive aspects of their units than nurses.

The proportion of nurses who tends to identify weaknesses in relation to the organization of services is much greater than other professionals.

In complementary education, for example, whereas there is one "No" for each 4 "Yes" assigned by physicians in this item, and almost one "No" for each three "Yes" assigned by dentists, among nurses this proportion was almost five, which was statistically significant (p = 0.0046). Career development programs was also another point on which this difference was very significant (p = 0.0000), where again, the proportion of nurses who reported the absence of or lack of participation in was much higher than other categories.

When a comparative analysis of the APS related to the models of care was conducted, the FHT with or without oral health predominated. Statistically significant differences were identified in career development program variables, where the proportion of professionals linked to the FHT, which has career development programs, was much smaller than the professionals integrated in other models of care (p=0.0000). Similarly, a statistically significant association regarding continuing education activities (p=0.0000) was observed, records of the documentation of cases referred for other services (p=0.0462), protocols to guide professionals for referrals to other services (p=0.0000) and use of complementary practices (p=0.0000). A significant difference was observed in the home visits, where the FHT presented a higher proportion of visits compared to the other two forms of attention (p=0.0000). 

**Table 3:** - Performance of primary care for patient access to the health system according professional category, Brazil, 2012

**Variables**	**Professional Category**												**P value**
	**Physician**				**Nurse**				**Dentist**				
	**Yes**	**%**	**No**	**%**	**Yes**	**%**	**No**	**%**	**Yes**	**%**	**No**	**%**	
Complementary education n=17202	800	4.6	193	1.1	13285	77.2	2591	15.1	264	1.5	69	0.4	0.0046*
Career development programs n=17113	303	1.8	670	4.0	3224	19.0	12412	73.3	98	0.6	229	1.4	0.0000*
Continuing education activities n=17113	853	5.0	132	0.8	12850	75.1	2951	17.2	254	1.5	73	0.4	0.0000*
All patients have their needs heard and assessed n=17047	956	5.6	25	0.15	15362	90.1	380	2.2	309	1.8	15	0.1	0.0384*
The team performs risk assessment during the intake n=13730	777	5.6	95	0.7	11066	80.6	1538	11.2	223	1.6	31	0.2	0.5189
Schedule is organized for home visitation n=11473	743	6.5	27	0.2	10013	87.3	480	4.2	201	1.8	9	0.1	0.3815
High risk patients are registered when referred n=13658	488	3.6	378	2.8	6261	45.9	6284	46.0	136	1.0	111	0.8	0.0004*
Form to register the patient referral n= 6885	377	5.5	111	1.6	5159	75.0	1102	16.0	107	1.6	29	0.4	0.0105*
Protocols that guide the prioritization of cases for referral n=13606	533	3.9	329	2.4	5797	42.6	6704	49.3	129	1.0	114	0.8	0.0000*
Regulation center for referral n=17047	905	5.3	76	0.4	14274	83.7	1468	8.6	292	1.7	32	0.2	0.2347
Forms for referral of patients n=17047	915	5.4	66	0.4	14029	82.3	1713	10.1	294	1.7	30	0.2	0.0001*
Sufficient medicines in primary care to meet population needs n=17015	606	3.6	373	2.1	10721	63.0	4992	30	205	1.2	118	0.7	0.0000*
Offering integrative and complementary practices n=17045	273	1.6	707	4.2	2865	16.8	12877	75.6	46	0.3	277	1.6	0.0000*
The team performs home visitation n=17045	977	5.7	4	0.02	15690	92.1	52	0.31	320	1.9	3	0.02	0.1846
The families of coverage area are frequently visited	927	5.5	50	0.3	14636	86.2	1054	6.2	289	1.7	31	0.2	0.0142*

* p <0,05

**Table 4:** - Performance of primary care for access to the patient according to the model of care, Brazil, 2012

**Activities**	**Model of care**												**P value**
	**FHT (with or without oral health)**				**Team AB**				**Other model**				
	**Yes**	**%**	**No**	**%**	**yes**	**%**	**No**	**%**	**Yes**	**%**	**No**	**%**	
Complementary education - V23 n= 17185	13883	80.8	2760	16.1	383	2.2	69	0.4	75	0.4	15	0.1	0.3059
Career development programs n=16923 v24	3516	21.0	12876	76.1	99	0.6	344	2.0	7	0.1	81	0.5	0.0000*
Continuing education activities = 17100 v25	13487	78.9	3074	18.0	283	2.2	66	0.4	80	0.5	10	0.1	0.0000*
All patients have their needs heard and assessed n=16987 v31	16055	94.6	397	2.3	422	2.5	15	0.1	85	0.5	3	0.0	0.1754
The team performs risk assessment during the intake n= 13723 v32	11710	85.3	1626	11.8	283	2.1	33	0.2	66	0.5	5	0.1	0.3987
Schedule is organized for home visitation n= 11473 v33	10678	93.1	486	4.2	236	2.1	22	1.32	43	0.4	8	0.1	0.3815
High risk patients are registered when referred n= 13658 v34	6685	50.0	6588	48.2	167	1.2	147	1.1	33	0.2	38	0.3	0.1323
Form to register the patient referral n= 6885 v35	5483	79.6	1202	17.5	136	2.0	31	0.5	24	0.4	9	0.1	0.0462*
Protocols that guide the prioritization of cases for referral n= 13606 v36	6289	46.2	6930	51.0	145	1.1	171	1.3	25	0.2	46	0.3	0.0000*
Regulation center for referral n= 17047 v37	12232	90.0	997	7.3	283	2.1	24	0.18	67	0.5	3	0.1	0.6982
Forms for referral of patients n= 17047	14782	86.7	1728	10.1	370	2.2	77	0.5	86	0.5	4	0.1	0.0000
V39 Has / receives medicines n= 17045	11146	59.5	5333	31.3	316	1.9	130	0.8	70	0.4	20	0.1	0.0286
V40 Offering integrative/ complementary practices n= 17045	3082	18.1	13426	78.8	93	0.6	354	2.1	9	0.1	81	0.5	0.0000*
V41 Team performs home visitation n = 17045	16462	96.6	46	0.3	437	2.6	10	0.1	88	0.5	2	0.1	0.0000*
V42 Families of coverage area are frequently visited n= 16987	15363	90.4	1099	6.5	404	2.4	33	0.2	85	0.5	3	0.1	0.1092

*p< 0,05

The Multiple Correspondence Analysis enabled the creation of the perceptual map shown in Figure 1, which demonstrates that the map can be divided into quadrants; on the right side, quadrants are plotted municipalities that showed better indicators in terms of qualification than those on the left.

**Figure 1:**
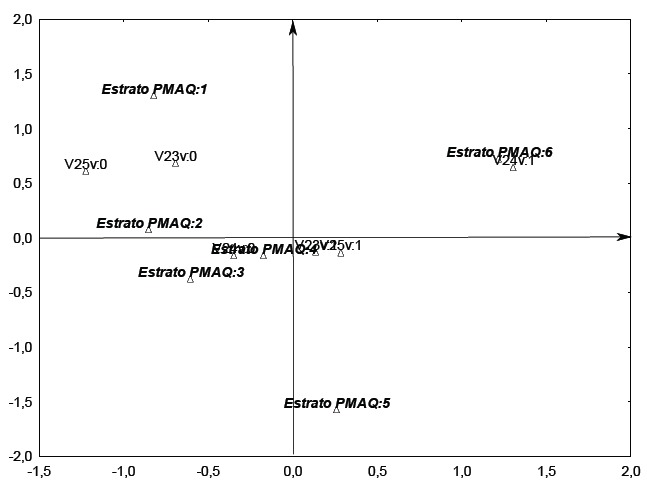
- Qualification for professionals working in the context of primary health care, according to the area of PMAQ, Brazil (2012)

Note: V23 Do you have or are you taking complementary education?; V24 Do you have career development programs?; V25 Are there continuing education activities in the municipality involving primary care professionals? Answers 1(Yes); 0 (No)

This figure demonstrate that the municipalities that comprise areas 5 and 6 present better indicators with regard to the training of their health professionals; the municipalities that are concentrated closer to the center have regular values. Thus they had some satisfactory indicators and others that were unsatisfactory, and municipalities of areas 1 and 2 had less satisfactory indicators for this item.

Figure 2 expresses the performance of municipalities in terms of availability, coordination of care, integration and supply using a perceptual map. On the right side of the map, the municipalities that showed better indicators are represented, and on the left side are those with poorer indicators.

Considering this evaluation with all of these attributes, the single area with satisfactory indicators across all of these dimensions was area 6; the municipalities of area 4 and 5 showed median values, with satisfactory indicators in some of those and unsatisfactory in others; however, the municipalities of area 5 were better than area 4; the municipalities of area 1, 2 and 3 did not achieve satisfactory results in these dimensions.

**Figure 2:**
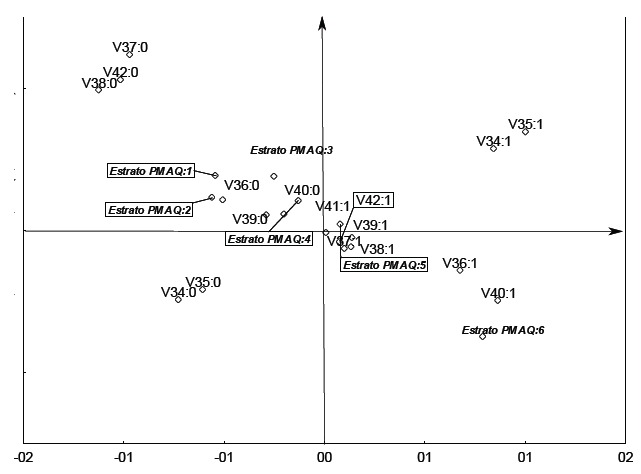
- Performance of municipalities for access to primary care according to the area defined by PMAQ, Brazil (2012)

Notes: V31 Do all patients coming to the clinic seeking care without appointments are heard and have their needs assessed?; V32 Does the team perform risk and vulnerability assessment during the intake of patients?; V33 Is the schedule organized to perform home visits?; COORDINATION OF CARE: V34 Does the staff keep records of the high risk patients referred to other points of care?; V35 IS there is form proving this?; V36 Are there, at the clinic, protocols to guide the prioritization of cases needing referral?; INTEGRATION: V37 Is there a central registration available for patient referral to other points of care? V38 Are there forms for patients referred to other points of care?; SUPPLIES: V39 Does the team have/receive enough basic medicines from the pharmacy to meet the needs of its population?; V40 Does the team offer service of complementary and integrative practices for patients of its area?; V41 Does the team perform home visits? V42 Are families in the coverage area of the primary care team frequently visited, according to risk and vulnerability assessments? Answers (1) Yes (0) No

Discussion

The prevailing participation of nurses as respondent in all area reveals their involvement with this level of assistance. In this sense, they are potentially able to cooperate with the UHC coverage by their role in all health care levels, and their particular desire to contribute to the achievement of the goal. The organization of nurses in international networks has been recognized by the PAHO/WHO, with an emphasis on achieving UHC and access to health care for the entire population ^(5)^.

In the assessment of the contextual or socioeconomic indicators and health, and the influence of professional qualification and territorial process in APS, areas 4, 5 and 6 showed better performance in all analyzed dimensions.

The best performance of the professional qualification in the present study, in areas 4, 5 and 6, was also observed in a study conducted in large cities, where more than half of physicians and nurses had participated in some training process in the prior 30 days^(15)^. 

Although a statistically significant difference was found between the areas with respect to career plan, all areas showed a weak performance in this item, which can be explained by the way in which professionals are recruitment. A study, conducted in Minas Gerais, showed that 75% of municipal health secretaries use temporary contracts for provision of services by professionals with higher education^(16)^.

This study highlights significant findings on the existence of continuing education actions. Continuing professional development is important, using information and communication technologies that facilitate the qualification of these professionals for the job. Such strategies also contribute to improving the problem solving within the FHU, and promote communication between specialists and generalists^(17)^.

With regard to coverage areas in Brazil, currently, the population coverage estimated by the APS teams becomes important as an universal indicator of success with the guidelines and goals of SUS^(18)^. It is necessary to note that, although the average number of persons under the responsibility of the team is within the recommendation of the Ministry of Health^(3)^, this number is considered high, if we consider that, in Brazil, the teams are responsible for a large number of activities^(19)^. 

To enable access to the population that is not covered by primary care, teams comply with the principle of universality, but also tend to undergo activity overloads, considering that more and more frequently the APS/FHT have new responsibilities delegated to them, and face responsibilities for diseases, priority groups, problems or specific situations^(20)^. A similar situation is seen in the UK and Europe, where professionals also develop a wide range of tasks, which include, among others: prevention activities, acute care/curative activities, treatment for patients with chronic conditions, and emergency treatment. These professionals are responsible for a roster of almost 2,250 people^(21)^.

Regarding availability, the unscheduled demand by patients to have their needs met and evaluated occurred in all areas, with better performance in areas 4, 5 and 6. These findings differ from those found by Giovanela, Fausto and Fidelis, which showed barriers to spontaneous demand and non-priority groups. Home visits are on the professional schedules in all areas of the municipalities. Similarly, this activity was observed as a routine of physicians and nurses in four large cities^(22)^. When comparing the models of care, there was a predominance of home visits being conducted by the FHT, a similar result to that found in a study with southern and northeastern cities^(10)^.

In the coordination of care, despite the significant differences between the areas, all areas presented unsatisfactory performance regarding the registration of referrals to other points of care, featuring a referral process without accountability and relationship with the patient.

In the integration of care, the existence of a central registration is present in the municipalities of the area analyzed, predominantly in 4, 5 and 6. Similar results were noted by physicians and nurses of the FHT that recognized the existence of a central registration for appointments and exams^(23)^.

With regard to the provision of health actions and services, there was a statistical significance in all aspects evaluated. The availability of medicines in the basic pharmacy to meet the population was observed in municipalities of all areas. In some cities of the country, this distribution is more related to priority groups^(15)^. It is remarkable to note the low supply of complementary and integrative practices for patients of the area, which may be linked to the fact that this type of care integrates a specialized service network, such as acupuncture offered in Porto Alegre^(24)^.

In the work process of the APS teams, the nurse takes on several assignments, among them: planning, individual and collective care, management, and systematic assessment of developed actions (PNAB. 20123), which may justify the tendency of nurses to negatively evaluate the actions of the organization. In the daily nursing work of the FHT units, difficulties occur, mainly related to lack of training for implementation of actions^(25)^. 

Regarding the contribution of nurses to universal access, the study showed that the majority were nurses, which shows in a way the involvement of this category of professional with the APS. The nurse has a more focused training for this area, with well-aligned curricula to the SUS social policy, with content in anthropology and sociology, health management, leadership and community sanitation practices, making her more sensitive to innovations in the context of the APS, and more motivated to promote change.

One important issue is that most nurses eventually assume leadership in the teams, strategically, and taking the forefront of primary care as a new mode of social production in health. The low pay of these professionals in the private sector makes many find the SUS to provide a chance for stability, which is very positive in terms of securing professionals in that category. One challenge is the establishment of a new model that values their core competence and recognizes their autonomy in prescribing and care. The hegemonic model with centrality in medical practice tends to push them out of this process.

Limitations

The study was not conducted in all the Brazilian municipalities, and only in those in which the teams voluntarily qualified for the PMAQ; thus, the results should be interpreted with caution because they do not retain the ability to be generalized. There is the possibility of selection bias, as not all staff members were included; only one staff member was chosen, and this was voluntary. Additionally, the study has design limitations, as it is a cross-sectional design, and is guided by interviews of professional. There was no monitoring of the teams for a period of time, or triangulation of data obtained from interviews with others, such as observation, records or statements of patients, which would increase the accuracy of the findings. However, it is important to note that the PMAQ is the first evaluation of this scope and methodological homogeneity and, despite the limitations, the findings contribute in the advancement of knowledge regarding APS-enhanced access, its critic nodes and also a situational diagnosis of which municipalities have advanced more in terms of universal coverage systems and those which have not.

Conclusion

The study showed that there is a relationship between access and socioeconomic conditions: as the area of the municipalities increases, the access to services tends to be better. However, within a context of social inequalities and iniquities, weaknesses are perceived that jeopardize the organization of health activities in the municipalities regarding the availability, care coordination, integration, and supply, particularly in the municipalities grouped in areas 1 to 3. Given the involvement of the nurse with the organization of health care, this professional has contributed to the potential access of APS in Brazil.

Now Read:

Research scenario

Adherence to cycle I of the PMAQ amounted to 17,482 Primary Care Teams (PCT), distributed across 3,944 (70.8%) of all cities and 14,111 Basic Health Units (BHUs)^(7)^. In this group, 17,202 were recruited for the study, as their questionnaires were validated in the database of the Ministry of Health.

Population and sample

The study population included professionals linked to the primary care team and qualified in PMAQ^(7)^, namely physicians, nurses, and dentists. In each team, only one sampling unit was selected for the study.

Measures and data sources

The questionnaires with closed-ended questions were provided on tablets, administered by interviewers who had the same training, under supervision. Next, they were sent online to the Ministry of Health system, accessed and validated by the higher education institution, based on a consistency analysis protocol and validation of the data collected through Validator's *online,* PMAQ-AB. The characteristics of the respondents and four (4) dimensions of the Module II questionnaire - Interview with professional of Primary Care Team and Document Checking of the Health Unit External Evaluation of the first cycle of the PMAQ-AB, were included here for data analysis^(7).^ The dimensions that were representative of the potential levels of access according to the authors' judgment were chosen and are described in the analysis plan.

Classification of cities according to the context variables

The cities listed in the study are classified into six groups, considering the per capita Gross Domestic Product (GDP), the percentage of the population with health insurance, the percentage of the population on the Bolsa Família (Family Grant) program, the percentage of the population in extreme poverty, and the population density.

The composition of the extracts considered for each municipality were: the lowest score among the percentage of the population with Bolsa Família program, and the percentage of the population in extreme poverty: group 1 - Cities with scores lower than 4.82 and a population of up to 10,000 inhabitants; group 2 - Cities with scores lower than 4.82 and a population of up to 20 thousand inhabitants; group 3 - cities with scores lower than 4.82 and a population of up to 50 thousand inhabitants; group 4 - Cities with scores between 4.82 and 5.40, and population of up to 100 thousand inhabitants; group 5 - Cities with scores between 5.4 and 5.85, and population of up to 500 thousand inhabitants; and cities with a score lower than 5.4, and population between 100.1 and 500 thousand inhabitants; and group 6 - Cities with population over 500,000 inhabitants, or a score equal or higher than 5.85^(7)^.

The variables under consideration to evaluate the potential access are described in Tables 1, 2 and 3 with dimensions, variables, their characteristic and nature:

Analysis plan

Initially, the descriptive analysis of the characteristics of the cities' groups was undertaken in terms of resources offered. Regarding the performance of the cities in terms of access, this was measured using four dimensions of the PMAQ tool: coverage group, supplies, customer coordination and integration. Therefore, the variables were dichotomized into yes and no, using the chi-square test of proportions to verify differences between the cities in relation to the size of potential access was used. The chi-square test with Yates' correction or Fisher's exact test was applied when necessary. For the population variable, the Kruskal-Wallis test was used to verify differences in relation to the median inhabitants monitored by group. In all tests applied, alpha was set at 5% (α = 0.05).

Ethical aspects

The multicenter project that led to the database was approved by the Research Ethics Committee at Universidade Federal do Rio Grande do Sul, under number 21904, on March 13^th^ 2012, and complied with the recommendations of National Health Council Resolution 196/1996 of the Ministry of Health.

Results

In total, 17,202 teams were recruited for the study, the majority being nurses (n = 15,876; 92.3%), with between one and three years of experience. In addition, 963 physicians participated in the study (5.6%) and 363 (2.1%) dentists with an equivalent length of experience.

Among the subjects enrolled, most professionals are affiliated with the Family Health Strategy (FHS) with oral health (n = 12,075; 70.2%). There was a median of one (1) physician, nurse, nursing technicians, and dentist per team. The data also reveal that 5991 (49.6%) participants could not answer whether the users covered by their unit could choose what health service to be followed at.

In Table 1, the performance of cities in terms of patient access is verified, considering the group established in PMAQ. Statistically significant differences were identified between the cities of groups 1, 2 and 3 with groups 4, 5 and 6, and the professionals of the last groups had more qualifications (p=0.0000). Regarding the career plan, statistically significant difference (p = 0.0000) were also observed, and the cities of group 4, 5 and 6 had better indicators; lowest values were found in groups 1, 2 and 3. Also, these groups showed statistically significant differences associated with their training and continuing education policy (p=0.0000).

According to Table 2, the performance indicators related to resource availability, coordination and integration capacity are highlighted. As observed, again, groups 4, 5 and 6 present better scores, with statistical significance, such as having the users' needs listened to (p=0.0000), welcoming with risk classification (p=0.0000) and organized agenda for home visits (p=0.0000). Records of complaints and team conducts for care coordination, as well as the existence of a registry system (p=0.0000) and the presence of an established regulation system (p=0.0000) were other aspects on which cities 4, 5 and 6 performed better.

Table 3 presents the cities' performance concerning the supply or list of services. Cities classified in groups 4, 5 and 6 presented better indicators in terms of sufficient drugs to attend to their population (p=0.0000). Nevertheless, a larger proportion of professionals in group 6 reported on the use of alternative or complementary health practices (p=0.0000).

**Table 1:** - Performance of cities concerning professional qualification and territorialization for users' access to the universal coverage systems by groups. Program for Better Access and Quality of Basic Care, Brazil, 2012.

**Dimension**	**Variables**	**Groups**						
		**1n (%)**	**2n (%)**	**3n (%)**	**4n (%)**	**5n (%)**	**6n (%)**	p value*
Professional Qualification	Do you have or are you taking a complementary degree (n=17,202)							0.000
	Yes	1,708 (9.93)	1,795 (10.43)	2,050 (11.92)	2,694 (15.66)	2,460 (14.30)	3,642 (21.17)	
	No	457 (2.66)	478 (2.78)	477 (2.77)	572 (3.33)	354 (2.06)	515 (2.99)	
	Is there a career plan (n=16,93.6)							0.000
	Yes	253 (1.49)	159 (0.94)	246 (1.46)	574 (3.39)	581 (3.43)	1,810 (10.69)	
	No	1,877 (11.08)	2,069 (12.22)	2,245 (13.26)	2,647 (15.63)	2,194 (12.95)	2,279 (13.46)	
	Are there continuing education actions involving basic care professionals (n=17,113)							0.000
	Yes	1,432 (8.37)	1,596 (9.33)	1,878 (10.97)	2,601 (15.20)	2,481 (14.50)	3,969 (23.19)	
	No	720 (4.21)	658 (3.85)	630 (3.68)	650 (3.80)	325 (1.90)	173 (1.01)	
Territorialization	How many people are under the team's responsibility							0.0001^?^
	Average	2165	2273	2527	3266	2814	4157	
	Were risk and vulnerability criteria considered to define the people under the team's responsibility (n=15,691)							0.0000
	Yes	1,024 (6.53)	1,141 (7.27)	1,323 (8.43)	1,705 (10.87)	1,423 (9.07)	2,648 (16.88)	
	No	951 (6.06)	877 (5.59)	937 (5.97)	1,265 (8.06)	1,115 (7.11)	1,282 (8.17)	
	Is the team's coverage group defined (n=17,150)							0.0000
	Yes	2,086 (12.16)	2,197 (12.81)	2,456 (14.32)	3,190 (18.60)	2,763 (16.11)	4,113 (23.98)	
	No	68 (0.40)	60 (0.35)	63 (0.37)	71 (0,41)	43 (0.25)	40 (0.23)	
	Is there population uncovered by basic care within the coverage group of the team (n=17,092)							0.0000
	Yes	369 (2.16)	534 (3.12)	888 (5.20)	1,083 (6.34)	1,391 (8.14)	1,513 (8.85)	
	No	1,783 (10.43)	1,724 (10.09)	1,618 (9.47)	2,170 (12.70)	1,406 (8.23)	2,613 (15.29)	
	How frequently does this team attend to people from outside the coverage group (n=16,855)							0.0000
	All weekdays	900 (5.34)	828 (4.91)	1,001 (5.94)	1,247 (7.40)	1,255 (7.45)	2,152(12.77)	
	Some weekdays	966 (5.73)	1,135 (6.73)	1,201 (7.13)	1,502 (8.91)	1,222 (7.25)	1,673 (9.93)	
	No weekdays	248 (147)	243 (1.44)	266 (1.58)	451 (2.68)	287 (1.70)	178 (1.65)	

* statistically significant p value (p<0.05). † Application of Kruskal-Wallis test. Source: Database of Program for Better Access and Quality of Basic Care - 1^st^ cycle, Ministry of Health, Brazil, 2012.

**Table 2:** - Performance of cities concerning the availability of resources, care coordination and integration capacity among services for user access to universal coverage systems by groups, Program for Better Access and Quality of Basic Care, Brazil, 2012.

**Dimension**	**Variables**	**Groups**						
		**1n (%)**	**2n (%)**	**3n (%)**	**4 n (%)**	**5n (5)**	**6n (%)**	p value*
Availability	Users who arrive spontaneously have their needs heard and assessed (n=17,140)							0.0000
	Yes	2,121 (12.37)	2,202 (12.85)	2,442 (14.25)	3,180 (18.55)	2,689 (15.69)	4,078 (23.79)	
	No	38 (0.22)	59 (0.34)	80 (0.47)	83 (0.48)	108 (0.63)	60 (0.35)	
	The team performs risk and vulnerability assessment in users welcoming (n=13,739)							0.0066
	Yes	1,265 (9.21)	1,385 (10.08)	1,645 (11.97)	2,286 (16.64)	2,050 (14.92)	3,442 (25.05)	
	No	192 (1.40)	221 (1.61)	248 (1.81)	324 (2.36)	236 (1.72)	445 (3.24)	
	The agenda is organized to make home visits (n=13,951)							0.0000
	Yes	1,418 (10.16)	1,628 (11.67)	1,865 (13.37)	2,391 (17.14)	2,253 (16.15)	3,697 (26.50)	
	No	134 (0.96)	115 (0.82)	114 (0.82)	149 (1.07)	104 (0.75)	83 (0.590)	
Care Coordination	Maintains registry of highest-risk users forwarded to other care services (n=17,104)							0.0000
	Yes	826 (4.83)	818 (4.78)	1,104 (6.45)	1,474 (8.62)	1,353 (7.91)	2,385 (13.94)	
	No	1,310 (7.66)	1,439 (8.41)	1,405 (8.21)	1,785 (10.44)	1,449 (8.47)	1,756 (10.27)	
	Are there documents that prove the coordination (n= 7,960)							0.0000
	Yes	605 (7.60)	638 (8.02)	913 (11.47)	1,206 (15.15)	1,132 (14.22)	1,978 (24.85)	
	No	221 (2.78)	180 (2.26)	191 (2.40)	268 (3.37)	221 (2.78)	407 (5.11)	
	Are there protocols to guide the prioritization of the cases that need forwarding (n=17,037)							0.0000
	Yes	581 (3.41)	613 (3.60)	807 (4.74)	1,213 (7.12)	1,228 (7.21)	2,907 (17.06)	
	No	1,558 (9.14)	1,636 (9.60)	1,685 (9.89)	2,036 (11.95)	1,567 (9.20)	1,206 (7.08)	
	Is there a regulation central (n=17,201)							0.0000
	Yes	1,880 (10.93)	2,006 (11.66)	2,239 (13.02)	2,907 (16.90)	2,540 (14.77)	4,027 (23.41)	
	No	284 (1.65)	267 (1.55)	288 (1.67)	359 (2.09)	274 (1.59)	130 (0.76)	
Integration	Is there a form for forwarding users to the other care services (n=17,201)							0.0000
	Yes	1,752 (10.19)	1,828 (10.63)	2,138 (12.43)	2,970 (17.27)	2,615 (15.20)	4,055 (23.57)	
	No	412 (2.40)	445 (2.59)	389 (2.26)	296 (1.72)	199 (1.16)	102 (0.59)	

*statistically significant p value (p<0.05). Source: Database of Program for Better Access and Quality of Basic Care - 1st cycle, Ministry of Health, Brazil, 2012.

**Table 3:** - Performance of cities concerning supply and list of services for user access to universal coverage systems according by groups, Program for Better Access and Quality of Basic Care, Brazil, 2012.

**Dimension**	**Variables**	**Groups**						
		**1n (%)**	**2n (%)**	**3n (%)**	**4n (%)**	**5n (%)**	**6n(%)**	p value*
Supply	Receives sufficient drugs from basic pharmacy to attend to its population (n=17,161)							0.0000
	Yes	1,459 (8.50)	1,490 (8.68)	1,722 (10.03)	2,210 (12.88)	1,830 (10.66)	2,898 (16.89)	
	No	378 (2.20)	457 (2.66)	614 (3.58)	644 (3.75)	718 (4.18)	2,077 (6.28)	
	Does not receive drugs	316 (1.84)	320 (1.86)	187 (1.09)	406 (2.37)	263 (1.53)	172 (1.00)	
	Offers integrative and complementary practices to users within the territory (n=17,199)							0.0000
	Yes	235 (1.37)	230 (1.34)	305 (1.77)	381 (2.22)	512 (2.98)	1,546 (8.99)	
	No	1,929 (11.22)	2,042 (11.87)	2,222 (12.92)	2,885 (16.77)	2,301 (13.38)	2,611 (15.18)	
	Performs home visits (n=17,199)							0.0075
	Yes	2,146 (12.48)	2,262 (13.15)	2,521 (14.66)	3,253 (18.91)	2,802 (16.29)	4,148 (24.12)	
	No	18 (0.10)	10 (0.06)	6 (0.03)	13 (0.08)	11 (0.06)	9 (0.05)	
	The families within the coverage group are visited periodically according to risk and vulnerability assessments (n=17,132)							0.0000
	Yes	1,963 (11.46)	2,069 (12.08)	2,345 (13.69)	2,997 (15.30)	2,621 (15.30)	3,986 (23.27)	
	No	183 (1.07)	193 (1.13)	176 (1.03)	256 (1.49)	181 (1.06)	162 (0.95)	

*Statistically significant p value (p<0.05). Source: Database of Program for Better Access and Quality of Basic Care - 1st cycle, Ministry of Health, Brazil, 2012.

Discussion

The prevailing participation of nurses as respondents in all groups reveals their involvement with PHC. The organization of nurses in international networks, recognized by the Pan American Health Organization, highlights this role for universal health coverage^(5)^. In the assessment of the influence of contextual indicators and health on professional qualification and territorialization, groups 4, 5 and 6 showed better performance with a larger population size and socioeconomic development. This reflects the unequal distribution of physicians and qualified nurses, a limiting factor of universal coverage^(3,13)^. This factor also happens in different countries, such as the United States, Australia^(13)^, Mexico, Ghana and Thailand^(3)^, China^(14)^. The strategies to attract and fix the professionals are context-based and multifaceted and their qualification in the course of their career stands out in the global scope^(13,15)^.

The PMAQ revealed qualification and continuing education strategies for the teams, combined with the use of information and communication technologies, which facilitate the qualification, improve the problem-solving ability and enhance the communication between general PHC practitioners and specialists^(16)^.

With regard to territorialization, each health team attends to an appropriate number of people. In Brazil, the territorialization gains further depth with the expanded coverage of the Family Health Strategy, following the logic supply-service-territory, despite the increasing flexibility of the territory for the population's needs, bonding and accountability. Nevertheless, planning based on the service logic ends up limiting the supply^(17)^.

On the one hand, Family Health takes form as a strategy towards universal coverage, including populations that used to be unattended. On the other hand, despite respecting parameters, the large number of people, the wide range of tasks, with promotion, prevention and treatment for priority groups, chronic illnesses, strategic situations of vulnerability put a strain on the professionals^(18)^. The Brazilian experience affirms that multiprofessional teamwork enhances the different dimensions of care in view of the expanded coverage^(19)^. This aspect concerning the greater impact of the primary care teams' interprofessional cooperation, particularly in cases of chronic illnesses, can be observed in the literature from other countries, clearly showing the need for clarifications on its potentials and limits^(20)^. 

In terms of availability, it is verified that the needs of the users who spontaneously visit the service are assessed and attended to in all groups, also with better performance for groups with larger populations. The Family Health initiatives to integrate the two types of demands - spontaneous and scheduled - represent one of the main challenges for access. There is a change from technical to user-centered care, the base of the PHC principles. Based on the international accumulation of lessons learned since the 1990's in Denmark and the United Kingdom, in 2005, the Institute of Medicine (IOM) launches a proposal to implement it by 2020 as one of the quality domains of the primary health care reform in the United States^(21)^.

In this study, the home visit is present on the agenda of professionals from cities in all groups. The home visits are fundamental for PHC and are a positive element of the access. Nevertheless, assessing their occurrence is not enough. Their impact on the health conditions and quality of the processes should also be assessed. In a research undertaken in Germany, it was revealed that the PHC professionals are in doubt on their efficacy, consider it as an obligation and do not feel motivated to make the visits^(22)^. This reflection reveals, for the Brazilian reality as well, the need to debate with the professionals on their effects and forms of incentive.

In the forwarding to other care points, despite significant differences between groups, it is observed that all groups present hardly satisfactory behavior, revealing difficulties in user accountability outside the BHS. Regulation centrals more frequently exist in the same groups highlighted earlier. These points reveal weaknesses in the coordination, continuity and integration of care at the different levels of network care. The Health Care Networks represent the Brazilian option to further the access and quality recommended by the Pan American Health Organization, as a way to fight the fragmentation and promote the integration of health systems in Latin America and the Caribbean. In these systems, despite particularities and complexities, a range of challenges is faced due to the coexistence of subsystems and different degrees of integration in the same system, besides structural issues^(23)^.

Concerning the supply of health actions and services, statistical significance was verified in the groups for all aspects assessed, including basic drugs. According to WHO, the systems that implemented the universal coverage need to address appropriate medication use, verify its benefits and avoid waste in order to guarantee sustainability^(24)^. The low level of integrative and complementary practices was verified for users on the territory, as the SUS has recommended since 2006. In addition, WHO reaffirms the importance of integrating scientific and traditional medicine for the purpose of global health^(25)^.

Limitations

The generalizability of the external evaluation committee of the first PMAQ cycle is limited because it did not cover all teams and worked with a statistically non-representative sample that, due to feasibility issues and/or the political nature of the assessment, presupposes the municipal health manager's voluntary adherence. Nevertheless, its unique range across the Brazilian territory with a homogeneous method, is undeniable. The cities' grouping reveals inequities in the supply, advances and critical knots among the groups of cities. The main limitation is that, because of its multifaceted nature, the object needs to be analyzed by parts. Based on the available data, the needs dimensions could not be assessed, nor could the effective use of the services and their impact on population health. The information was based on "done/not done" answers, and further depth is needed as to how the actions are being accomplished and their appropriateness to the demands and quality parameters. Other studies are needed, using multiple methods capable of articulating quantitative data with qualitative case studies, with a view to better apprehending the complexity of the object.

Conclusions

The study showed that there is a relationship between access and socioeconomic conditions: as the group of the cities increases, the access to services tends to be better. However, within a context of social inequalities and iniquities, weaknesses are perceived that jeopardize the organization of health activities in the cities regarding the availability, care coordination, integration, and supply, particularly in the cities grouped in groups 1 to 3. Given the involvement of the nurse with the organization of health care, this professional has contributed to the potential access to PHC in Brazil. The curricula for work in this group are aligned with the social policies of the SUS, which include contents on anthropology, sociology, health management, leadership and health practices in the communities. This factor makes the nurses more porous to innovations and team leaderships in the PHC context, with greater motivation to promote changes, as opposed to low remuneration in the private sector. Their engagement entails the challenge of recognition for nursing competencies and autonomy in prescription and in care not exclusive to the medical category.

References

1. Organização Pan-Americana da Saúde. Estratégia para o acesso universal à saúde e cobertura universal de saúde. [Internet]. Washington, D.C: OPS; 2014. [Acesso 12 nov 2014]. Disponível em: www.paho.org/hq/index.php?option=com_docman&task

2. Rodin J. Accelerating action towards universal health coverage by applying a gender lens. Bull Wrld Health Org. 2013; 91:710-711. doi:http://dx.doi.org/10.2471/BLT.13.127027.

3. Ministério da Saúde (BR). Secretaria de Atenção à Saúde. Departamento de Atenção Básica. Política Nacional de Atenção Básica [Internet]. Brasília: Ministério da Saúde; 2012. [Acesso 8 nov 2013]. 110 p. (Série E. Legislação em Saúde). Disponível em: http://dab.saude.gov.br/portaldab/biblioteca.php?conteudo=publicacoes/pnab. 

4. Victora CG, Wagstaff A, Schellenberg JA, Gwatkin D, Claeson M, Habicht JP. Applying an equity lens to child health and mortality: more of the same is not enough. Lancet. 2003; 362(9379):233-41.

5. Cassiani SHDB. Estratégia para o acesso universal à saúde e cobertura universal de saúde e a contribuição das Redes Internacionais de Enfermagem. Rev. Latino-Am. Enfermagem. 2014; 22(6):891-2

6. Molina J. Para não perder o trem da história!. Rev esc enferm USP. [Internet]. 2014 [Acesso 22 maio 2015]; 48(1):8-17. Disponível em: http://www.scielo.br/scielo.php?script=sci_arttext&pid=S0080-62342014000100008&lng=en. http://dx.doi.org/10.1590/S0080-623420140000100001.

7. Ministério da Saúde (BR). Portaria n. 1.654, de 19 de julho de 2011 (BR). Institui, no âmbito do Sistema Único de Saúde, o Programa Nacional de Melhoria do Acesso e da Qualidade da Atenção Básica (PMAQ-AB) e o Incentivo Financeiro do PMAQ-AB, denominado Componente de Qualidade do Piso de Atenção Básica Variável - PAB Variável. Diário Oficial [da] República Federativa do Brasil, Brasília: 20 jul. 2011. n. 138, Seção I, p. 79.

8. Noronha JC. Cobertura universal de saúde: como misturar conceitos, confundir objetivos, abandonar princípios. Cad Saúde Pública. [Internet]. 2013. [Acesso 15 mar 2015]; 29 (5): 847-9. Disponível em: http://dx.doi.org/10.1590/S0102-311X2013000500003. 

9. Cardoso MO, Vieira-da-Silva LM. Avaliação da cobertura da atenção básica à saúde em Salvador, Bahia, Brasil (2000 a 2007). Cad Saúde Pública. [Internet]. jul 2012 [Acesso 14 jan 2015]; 28(7):1273-84. Disponível em: http://www.scielosp.org/scielo.php?script=sci_arttext&pid=S0102-311X2012000700006&lng=pt&nrm=iso&tlng=pt. 

10. Tomasi E, Facchini LA, Thumé E, Piccini RX, Osorio A, Silveira DS, et al. Características da utilização de serviços de Atenção Básica à Saúde nas regiões Sul e Nordeste do Brasil: diferenças por modelo de atenção. Ciênc Saúde Coletiva. 2011;16(1):4395-404. 

11. Leão C, Caldeira AP. Avaliação da associação entre qualificação de médicos e enfermeiros em atenção primária em saúde e qualidade da atenção. Ciênc Saúde Coletiva. 2011; 16( 11 ): 4415-4423.

12. Taddeo PS, Gomes KWL, Caprara A, Gomes AMA, Oliveira GC, Moreira TMM. Acesso, prática educativa e empoderamento de pacientes com doenças crônicas. Ciênc Saúde Coletiva. 2012;17(11): 2923-30.

13. Spencer, NH. Essentials of Multivariate data Analysis. CRC. PRESS: Taylor & Francis; 2014. 186 p.

14. Mingoti SA. Análise de dados através de métodos de es- tatística multivariada: uma abordagem aplicada. Belo Horizonte: Editora UFMG; 2005.

15. Almeida PF, Fausto MCR, Giovanella L. Fortalecimento da atenção primária à saúde: estratégia para potencializar a coordenação dos cuidados. Rev Panam Salud Publica. 2011; 29(2):84-95.

16. Junqueira TS, Cotta RMM, Gomes RCG, Silveira SFR, Siqueira-Batista R, Pinheiro TMM, Sampaio RF. As relações laborais no âmbito da municipalização da gestão em saúde e os dilemas da relação expansão/precarização do trabalho no contexto do SUS. Cad Saúde Pública. 2010; 26(5):918-28. 

17. Giovanella L, Mendonça MHM, Almeida PF, Escorel S, Almeida PF, Fausto MCR, et al. Potencialidades e obstáculos para a consolidação da Estratégia Saúde da Família em grandes centros urbanos. Saúde em Debate. 2010;34(85):248-64.

18. Ministério da Saúde (BR). Secretaria de Gestão Estratégica e Participativa. Departamento de Articulação Interfederativa. Caderno de Diretrizes: Objetivos, Metas e 32 Indicadores 2013 - 2015 [Internet]. Brasília: 2013 [Acesso 29 nov 2013]. 156 p. (Série Articulação Interfederativa, v. 1). Disponível em: http://portalweb04.saude.gov.br/sispacto/Caderno.pdf. 

19. Souza MB; Rocha PM; Sá AB; Uchoa SAC. Trabalho em equipe na atenção primária: a experiência de Portugal. Rev Panam Salud Publica. [Internet]. mar 2013;33(3):190-5. [Acesso 20 out 2013]. Disponível em: http://dx.doi.org/10.1590/S1020-49892013000300005.

20. Tesser, CD, Norman AH. Repensando o acesso ao cuidado na Estratégia Saúde da Família. Saúde soc. São Paulo. 2014; 23(3):869-83. doi.org/10.1590/S0104-12902014000300011.

21. Calnan M, Hutten J, Tiljak H. The challenge of coordination: the role of primary care professional in promoting integration across the interface. In: Saltman RS, Rico A, Boerma WGW, editores. Primary care in the driver's seat? Organizational Reform in European Primary Care [Internet]. Berkshire: Open University Press; 2007 [Acesso 12 jun 2014]. p. 85-104. Disponível em: http://www.euro.who.int/__data/assets/pdf_file/0006/98421/E87932.pdf. 

22. Santos AM, Giovanella L, Mendonça MHM, Andrade CLT, Maria Inês Carsalade Martins, Cunha MS. Praticas assistenciais das equipes de saúde da família em quatro grandes centros urbanos. Ciênc Saúde Coletiva. 2012;17(10):2687-702.

23. Almeida Patty Fidelis de, Giovanella Lígia, Mendonça Maria Helena Magalhães de, Escorel Sarah. Desafios à coordenação dos cuidados em saúde: estratégias de integração entre níveis assistenciais em grandes centros urbanos. Cad Saúde Pública. [Internet]. fev 2010 [Acesso 30 maio 2015]; 26(2):286-98. Disponível em: http://www.scielo.br/scielo.php?script=sci_arttext&pid=S0102-311X2010000200008&lng=en. http://dx.doi.org/10.1590/S0102-311X2010000200008.

24. Dallegrave D, Camila Boff C , Kreutz JA. Acupuntura e Atenção Primária à Saúde: análise sobre necessidades de usuários e articulação da rede. Rev Bras Med Fam Comunidade. 2011; 6(21):249-56.

25. Brondani DA Jr, Heck RM, Ceolin T, Viegas CRS. Atividades gerenciais do enfermeiro na estratégia de saúde da família. Rev Enferm UFSM. 2011;1(1):41-50.

References

1. Abiiro GA, De Allegri M. Universal health coverage from multiple perspectives: a synthesis of conceptual literature and global debates. BMC Int Health Hum Rights. 2015;15(17):1-7. 

2. Rodin J. Accelerating action towards universal health coverage by applying a gender lens. Bull World Health Organ. 2013;91(9):710-11. 

3. Campell J, Buchan J, Cometto G, David B, Dussault G, Fogstad H et al. Human resources for health and universal health coverage: fostering equity and effective coverage. Bull World Health Organ. 2013;91:853-63.

4. Victora CG, Wagstaff A, Schellenberg JA, Gwatkin D, Claeson M, Habicht JP. Applying an equity lens to child health and mortality: more of the same is not enough. The Lancet. 2003;362(1):233-41.

5. Cassiani SHDB. Strategy for universal access to health and universal health coverage and the contribution of the International Nursing Networks. Rev. Latino-Am. Enfermagem. 2014;22(6):891-2.

6. Molina J. Para não perder o trem da história! Rev. esc. enferm. USP. 2014;48(1):8-17. 

7. Pinto HA, Sousa ANA, Ferla AA. O Programa Nacional de Melhoria do Acesso e da Qualidade da Atenção Básica: várias faces de uma política inovadora. Saúde debate. 2014;38(spe):358-72. 

8. Noronha JC. Cobertura universal de saúde: como misturar conceitos, confundir objetivos, abandonar princípios. Cad. saúde pública. 2013;29(5):847-9.

9. Cardoso MO, Vieira-da-Silva LM. Avaliação da cobertura da atenção básica à saúde em Salvador, Bahia, Brasil (2000 a 2007). Cad. Saúde Pública. 2012;28(7):1273-84. 

10. Tomasi E, Facchini LA, Thumé E, Piccini RX, Osorio A, Silveira DS, et al. Características da utilização de serviços de Atenção Básica à Saúde nas regiões Sul e Nordeste do Brasil: diferenças por modelo de atenção. Ciênc. saúde coletiva. 2011;16(1):4395-404. 

11. Leão C, Caldeira AP. Avaliação da associação entre qualificação de médicos e enfermeiros em atenção primária em saúde e qualidade da atenção. Ciênc. saúde coletiva. 2011;16(11):4415-23.

12. Taddeo PS, Gomes KWL, Caprara A, Gomes AMA, Oliveira GC, Moreira TMM. Acesso, prática educativa e empoderamento de pacientes com doenças crônicas. Ciênc. saúde coletiva. 2012;17(11):2923-30.

13. Oliveira FP, Vanni T, Pinto HA, Santos JTR, Figueiredo AM, Araújo SQ et al. Mais Médicos: um programa brasileiro em uma perspectiva internacional. Interface (Botucatu). 2015;19(54):623-34.

14. Wang X, Zheng A, He X, Jiang H. Integration of rural and urban healthcare insurance schemes in China: an empirical research. BMC Health Serv Res. 2014;14(42):1-10.

15. Huicho L, Dieleman M, Campbell J, Codjia L, Balabanova D, Dussault G, et al. Increasing access to health workers in underserved areas: a conceptual framework for measuring results. Bull World Health Organ. 2010;88(5):357-63.

16. Giovanella L, Mendonça MHM, Almeida PF, Escorel S, Almeida PF, Fausto MCR, et al. Potencialidades e obstáculos para a consolidação da Estratégia Saúde da Família em grandes centros urbanos. Saúde em Debate. 2010;34(85):248-64.

17. Faria RM. A Territorialização da Atenção Primária à Saúde no Sistema Único de Saúde e a construção de uma perspectiva de adequação dos serviços aos perfis do território urbano. Hygeia. 2013;9(16):121-30.

18. Souza MB; Rocha PM; Sá AB; Uchoa SAC. Trabalho em equipe na atenção primária: a experiência de Portugal. Rev Panam Salud Publica. 2013;33(3):190-5

19. Tesser, CD, Norman AH. Repensando o acesso ao cuidado na Estratégia Saúde da Família. Saúde soc. São Paulo. 2014;23(3):869-83. 

20. Morgan S, Pullon S, McKinlay E. Observation of interprofessional collaborative practice in primary care teams: An integrative literature review. Int J Nurs Stud. 2015;52(7):1217-30.

21. Davis K, Schoenbaum SC, Audet AM. A 2020 Vision of Patient-Centered Primary Care. J Gen Intern Med. 2005;20(10):953-57. 

22. Theile G, Kruschinski C, Buck M, Müller CA, Hummers-Pradier E. Home visits - central to primary care, tradition or an obligation? A qualitative study. BMC Fam Pract. 2011;12(24):1-11.

23. Ramagem C, Urrutia S, Griffith T, Cruz M, Fabrega R, Holder R, et al. Combating health care fragmentation through integrated health services delivery networks. Int J Integr Care. 2011;11(Suppl):1-2. 

24. Wagner AK, Quick JD, Ross-Degnan D. Quality use of medicines within universal health coverage: challenges and opportunities. BMC Health Serv Res. 2014;14(357):1-6.

25. Falkenberg T, Smith M, Robinson N. Traditional and integrative approaches for global health. Eur J Integr Med. 2015;7(1):1-4.

